# Editorial: Reimagining universal health coverage and other global health targets in the post-COVID-19 era

**DOI:** 10.3389/fpubh.2022.1070399

**Published:** 2023-01-04

**Authors:** Vijay Kumar Chattu, Behdin Nowrouzi-Kia, Thankam Sunil, Hamid Allahverdipour

**Affiliations:** ^1^Department of Occupational Science and Occupational Therapy, Temerty Faculty of Medicine, University of Toronto, Toronto, ON, Canada; ^2^Center for Interdisciplinary Research, Saveetha Dental College, Saveetha Institute of Medical and Technical Sciences (SIMATS), Saveetha University, Chennai, India; ^3^Department of Community Medicine, Faculty of Medicine, Datta Meghe Institute of Medical Sciences, Wardha, India; ^4^Department of Public Health, University of Tennessee, Knoxville, Knoxville, TN, United States; ^5^Research Center of Psychiatry and Behavioral Sciences, Tabriz University of Medical Sciences, Tabriz, Iran; ^6^Department of Health Education and Promotion, Tabriz University of Medical Sciences, Tabriz, Iran

**Keywords:** universal health coverage, Sustainable Development Goal (SDG), COVID-19, pandemic, global health security, health systems strengthening, risk perception

This special issue, “Reimagining Universal Health Coverage and other global health targets in the post-COVID-19 era,” presents a collection of articles on Universal Health Coverage (UHC) and other health-related United Nations' Sustainable Development Goals (SDGs). Achieving UHC was one of the world's nations' goals when they adopted the SDGs in 2015. UHC is firmly based on the 1948 WHO Constitution, which declares health a fundamental human right and commits to ensuring the highest attainable level of health for all (1). UHC means all individuals and communities have access to the necessary healthcare services without experiencing financial hardship. It encompasses the entire gamut of critical, high-quality health services, including health promotion, prevention, treatment, rehabilitation, and palliative care across the lifespan (1). Over 18 million additional health workers are required by 2030 to achieve the SDGs and UHC targets for the health workforce.

The special issue has a rich collection of 7 original research articles, 3 policy briefs, and 2 perspectives submitted globally from North America, Asia, Europe, and the Middle East, which included analyses of African and Caribbean regions. A study by Bergmann and Wagner addressed the impact of COVID-19 on informal caregiving across Europe from 26 countries based on the eighth wave of the Survey of Health, Aging, and Retirement in Europe (SHARE). The study has highlighted that the perception of unmet care needs was significantly associated with country differences regarding the duration of the stay-at-home orders and further called for a reduction of burden and symptoms of anxiety or depression for caregivers and care recipients.

Another study by Qazi et al., using Gray Incidence Analysis Model (Gray Relational Analysis), evaluated the health systems at the country level. They reported that the healthcare system of advanced countries, i.e., the UK, USA, France, Denmark, etc. (almost the whole of western Europe/Schengen area/OECD), has a very poor response to the shock of the COVID-19 pandemic, which is in contrast to the myth that these countries have the best healthcare systems in the world. However, 30 countries are categorized as countries having much better health systems, most of which are member countries of the Southern Africa Development Community. Another interesting study from Italy by Blasi et al., to renovate the current healthcare system and guarantee equal access to health services, have proposed a multidisciplinary Think Tank and proposed a manifesto with six drivers for change: vision, governance, competence, intelligence, humanity, and relationship. Further, each driver was linked to action to actively move toward a new healthcare system based on trust between science, citizens, and institutions.

Some of the articles have emphasized the role of health systems strengthening and, in this context, Cuschieri et al. have highlighted the Cypriot resilience plan in response to the lessons learned from the pandemic put forward by the Government of the Republic of Cyprus with 6% of the total budget (74.1 million euros) to be allocated on strengthening the capacity of the Global Health Security and supporting public health protection. They further called for the Cypriot government and other states with a similar population or geographical distribution to consider the transformation for public health emergency preparedness and transition to a working syndemic model. Similarly, a Mexican study by Ramos Herrera et al., have highlighted the timely installation and work of the University of Guadalajara- health situation room helped the state of Jalisco in Mexico to maintain one of the lowest incidence and mortality rates in the country.

Globally, the COVID-19 pandemic had several impacts on various dimensions, including the global economy. An in-depth analysis by Tang et al. on the impact of the UHC Healthcare system on stock returns during COVID-19 found that the sudden onset of an epidemic disease results in unevenly distributed medical system resources, consequently diminishing the impact of UHC on abnormal returns. Further, the study concluded that abnormal cumulate returns emerge at the early stages of the pandemic, signifying that the strategy of investment as a sudden reaction to the outbreak is normally at the beginning of the pandemic and that a well-organized UHC system is a key factor in avoiding the risk of damage to stock markets as a result of a sudden outbreak. Da Silva and Da Silva have analyzed the relationship between the country's gross domestic product and COVID-19 mortality globally and have reported various scenarios. Their statistical analysis did not reveal any relationship between GDP per capita and the COVID-19 mortality rate. With a base GDP-per capita level of US$1200, a significant statistical relationship (at 5%level) between GDP per capita and COVID-19 mortality rate can no longer be found (*p* = 0.0588).

An epidemiological study of COVID-19 in Saudi Arabia using the data from the Ministry of Health, as reported by Salam et al., has highlighted that though COVID-19 transmission since March 2020 is considered to be widespread, creating an excess burden on the public health system, the disciplined life in compliance with law and order paved the way for effective program implementation and epidemic control. In terms of the dynamics between urbanization and infectious disease spread, a Chinese national study by Yu et al., have concluded that urban education, employment and entrepreneurship, housing, medical and health care, and other basic public services brought by urbanization can help reduce the risk of the spread of infectious diseases. However, the increasing density of buildings caused by land urbanization increases the risk of spreading infectious diseases. Another study in China by Si et al. investigated the links between the COVID-19 vaccination and public attitudes toward protective countermeasures have found that gender, age, education level, occupation risk, individual health risk perception, public health risk perception, social responsibility, peer effect, and government supervision are the main drivers for participants to be vaccinated. The results further show that vaccination lessened participants' frequency of hand washing by 1.75 times and their compliance frequency intensity of observing physical distancing by 1.24 times. However, the rate of mask-wearing did not reduce significantly, implying that China's main countermeasure of effective mask-wearing effectively controls COVID-19.

UHC demands a multifaceted strategy. Primary health care and life course approaches are crucial. A primary healthcare strategy focuses on organizing and developing health systems so individuals can obtain services for their health and wellbeing based on their needs and preferences as early as possible and in their everyday settings. Two aspects should be considered when tracking progress toward UHC: (1) The proportion of a population that has access to basic, high-quality health care (SDG 3.8.1) and (2) The proportion of the population that spends a significant portion of their family income on health (SDG 3.8.2). In this regard, Frank et al. have highlighted that Canada ranks highest in the world on the UHC Service Coverage Index, at 89 on a scale of 0 to 100, surpassing comparator countries such as Australia, New Zealand, Norway, the UK, Netherlands, Sweden, and other G-7 nations including the USA, France, Germany. However, they emphasized that UHC systems could easily fall prey to powerful and wealthy forces worldwide, seeking to make healthcare just another profitable business commodity citing the example of “Cambie Trial” (Cambie Surgeries Corporation vs. British Columbia). In this context, Montagu shared the European experience in involving the private sector in achieving UHC, where it can be effectively provided with or without large-scale private sector provision in hospital, specialty, and primary care services, and moreover, it can be provided with high levels of patient satisfaction. The study further claimed that the European examples provide critical insight for governments of low-and-middle-income countries that large-scale privately provided medical services are neither necessary for achieving UHC nor a barrier to it. A recent article by Lal et al. highlighted that Health systems designed for UHC had been shown to support communities more equitably through primary health care (2).

In conclusion, the nations and states that progress toward achieving UHC will certainly make progress toward other health-related targets and goals. Good health allows children to learn and adults to earn, helps people escape from poverty and provides the basis for sustainable economic development. Therefore, this special issue provides original research, reviews, and evidence-based policy recommendations from various geographical regions in achieving the target of UHC amid the COVID-19 pandemic. The COVID-19 pandemic highlighted the critical significance of the health and care workforce and the importance of increasing spending in this sector to reap the economic benefits at the national level.

## Author contributions

VC wrote the first draft. BN-K, TS, and HA revised and provided critical inputs. All authors discussed the results, and recommendations and contributed their inputs to the final manuscript.
